# Prediction of disease flare by biomarkers after discontinuing biologics in patients with rheumatoid arthritis achieving stringent remission

**DOI:** 10.1038/s41598-021-86335-7

**Published:** 2021-03-25

**Authors:** Hideto Kameda, Ayako Hirata, Takaharu Katagiri, Yuto Takakura, Yuki Inoue, Sayaka Takenaka, Hideki Ito, Kennosuke Mizushina, Takehisa Ogura

**Affiliations:** Division of Rheumatology, Department of Internal Medicine, Faculty of Medicine, Toho University, 2-22-36 Ohashi, Meguro-ku, Tokyo, 153-8515 Japan

**Keywords:** Biomarkers, Medical research, Rheumatology

## Abstract

To elucidate the disease-flare process in rheumatoid arthritis (RA) after discontinuing biological disease-modifying antirheumatic drugs (bDMARDs), we first focused on RA-flare prediction after achieving stringent remission criteria. Patients with RA who maintained a simplified disease activity index ≤ 3.3 for ≥ 3 months during November 2014–January 2018 in our medical centre in Tokyo, Japan, were eligible. The primary endpoint was flare (disease activity score 28—erythrocyte sedimentation rate ≥ 3.2 with increase from baseline > 0.6) within 2 years after bDMARD discontinuation. Comprehensive clinical assessments, ultrasonographic evaluation of 40 joints, and blood sampling for 12 biomarkers were performed every 2–3 months for 2 years unless patients experienced flare. Flare-positive and flare-negative patients were compared using univariate and Kaplan–Meier analyses. Thirty-six patients (80.6% female, median disease duration, 5.2 years; median treatment period with discontinued bDMARD, 2 years; median remission duration, 18 months) were enrolled. Twenty patients (55.6%) experienced RA flare 43–651 (median, 115) days after the first skipped date of bDMARDs. Two patients who withdrew without disease flare were excluded from the comparison. Clinical and ultrasonographic evaluations did not show significant between-group differences; Kaplan–Meier analysis showed that higher baseline soluble tumour necrosis factor receptor 1 (sTNFR1) concentration impacted subsequent disease flare (p = 0.0041); higher baseline interleukin (IL)-2 concentration was exclusively beneficial to patients with lower sTNFR1 (p = 0.0058), resulting in remission maintenance in 83.3% of patients with lower sTNFR1 and higher IL-2. We demonstrated the usefulness of combined biomarker evaluation for predicting sustained remission after bDMARD discontinuation in RA.

## Introduction

Sustained remission has become a realistic treatment target for patients with rheumatoid arthritis (RA)^1^. Clinical remission defined by the stringent criteria of the American College of Rheumatology (ACR)/the European League Against Rheumatism (EULAR)^2^, namely the Boolean remission criteria in which patients must achieve a tender joint count (TJC) of all 28 joints (TJC28) ≤ 1, swollen joint count (SJC) of 28 joints (SJC28) ≤ 1, patient global assessment of disease activity (PtGA) ≤ 1 (0–10 cm = 0–100 mm visual analogue scale [VAS]) and serum C-reactive protein (CRP) ≤ 1 mg/dL, or simplified disease activity index^3^ (SDAI; the sum of TJC28, SJC28, PtGA [cm], physician or other evaluators’ global assessment of disease activity [PhGA; cm] and CRP [mg/dL]) ≤ 3.3, has been actually achieved by a considerable portion of patients in recent clinical trials examining biological disease-modifying antirheumatic drugs (bDMARDs)^4,5^, as well as in observational studies^6,7^.

The pathogenesis of RA has not been fully elucidated partly because of difficulty in the evaluation and follow-up of the population before RA development^8^. Instead, the process of RA flare, which may mimic that of RA development, can be closely monitored. The economic burden of bDMARDs may justify their discontinuation when patients achieve clinical remission or low disease activity^1^, and evidence of bDMARD discontinuation in such patients has accumulated^9–26^. Importantly, many patients successfully regained remission or low disease activity by re-treatment with the same bDMARD when they experienced disease flare after bDMARD discontinuation in the above studies.

Therefore, this prospective study was conceived to elucidate the process of RA flare by sequential assessment of clinical, imaging, and biomarker parameters. In this first report, we focused on the prediction of RA flare after bDMARD discontinuation by assessing the above-mentioned parameters starting at the point of achieving ACR/EULAR-defined clinical remission.

## Results

### Demographic and clinical characteristics of enrolled patients

Demographic and clinical characteristics of the 36 patients are summarised in Table 1. Two patients did not complete the 2-year study, one due to sudden relocation on day 43 and the other because of death due to comorbidity (cardiomyopathy) on day 175; neither had experienced disease flare at the time of drop out. Thus, these patients were excluded from the comparative study between flare-positive and flare-negative patients. Twenty-nine (80.6%) patients were female, and the median (interquartile range [IQR]) age at enrolment was 69 (53–75) years. The median (IQR) disease duration was 5.2 years, treatment with the discontinued bDMARD had lasted for 2.1 (1.4–3.5) years, and the median (IQR) remission duration was 20 (9–31) months (< 6 months in only two patients). Most (72.2%) patients had been under treatment with first bDMARD and 76.5% and 77.8% of the patients were respectively positive for anti-cyclic citrullinated peptide antibody (anti-CCP) and rheumatoid factor.

**Table 1:** Patient demographics and baseline clinical features.

	Total, n = 36	Flare ( +), n = 20	Flare (−), n = 14	p-value
Female, n (%)	29 (80.6)	16 (80.0)	12 (85.7)	1.00^b^
Age (years)	69 (53–75)	69 (48–76)	69 (54–75)	0.90^a^
Height (cm)	156 (152–160)	156 (152–160)	159 (154–160)	0.25^a^
Weight (kg)	50 (48–57)	50 (45–57)	51 (50–57)	0.32^a^
RA duration (years)	5.2 (2.3–9.8)	5.7 (2.5–9.8)	4.1 (1.6–9.7)	0.46^a^
RA onset to bDMARDs (years)	1.3 (0.4–7.8)	2.6 (0.5–7.1)	0.6 (0.4–5.7)	0.24^a^
bDMARD continuation (years)	2.1 (1.4–3.5)	1.9 (1.5–2.8)	2.7 (0.9–3.6)	0.83^a^
Remission duration (months)	20 (9–31)	18 (10–26)	24 (9–37)	0.42^a^
**Failed bDMARDs, n (%)**				
0	26 (72.2)	14 (70.0)	10 (71.4)	
1	8 (22.2)	5 (25.0)	3 (21.4)	0.95^b^
2	2 (5.6)	1 (5.0)	1 (7.1)	
Radiographic stage I or II, n (%)	27 (75.0)	17 (85.0)	10 (71.4)	0.41^b^
Anti-CCP positive, n (%)	26 (76.5)	16 (84.2)	8 (61.5)	0.22^b^
Rheumatoid factor positive, n (%)	28 (77.8)	17 (85.0)	9 (64.3)	0.23^b^
Rheumatoid factor titre (IU/mL)	43.6 (15.4–110.4)	59.7 (16.2–115.1)	24.4 (6.8–62.5)	0.18^a^
Tender joint count of 28 joints	0 (0–0)	0 (0–0)	0 (0–0)	1.00^a^
Swollen joint count of 28 joints	0 (0–0)	0 (0–1)	0 (0–0)	0.19^a^
Patient global assessment (/100)	3 (0–6)	3 (0–8)	3 (1–6)	0.76^a^
Physician global assessment (/100)	0 (0–3)	1 (0–5)	0 (0–2)	0.30^a^
HAQ-DI	0 (0–0)	0 (0–0)	0 (0–0.2)	0.71^a^
CRP (mg/dL)	0.04 (0.01–0.09)	0.04 (0.01–0.1)	0.04 (0.02–0.06)	0.83^a^
ESR (mm/ 1 h)	16 (6–27)	20 (8–35)	9 (5–18)	0.077^a^
Matrix metalloproteinase-3 (ng/mL)	53.8 (22.6–89.3)	57.1 (45.2–137.9)	50.5 (44.4–68.0)	0.24^a^
DAS28-ESR	2.0 (1.3–2.4)	2.3 (1.4–2.8)	1.6 (1.1–2.1)	0.058^a^
SDAI	0.4 (0.2–1.5)	0.6 (0.3–2.1)	0.4 (0.1–1.2)	0.45^a^
ACR/EULAR Boolean remission, n (%)	31 (86.1)	17 (85.0)	12 (85.7)	1.00^b^
Ultrasound grey-scale	7 (1–10)	6 (1–12)	8 (3–10)	0.54^a^
Ultrasound Power Doppler	0 (0–1)	0 (0–1)	0 (0–1)	0.88^a^
Ultrasound total score	7 (1–12)	6 (1–13)	8 (3–11)	0.53^a^
**Discontinued bDMARDs**				
TNF inhibitors, n (%)	26 (72.2)	14 (70.0)	12 (85.7)	0.42^b^
Adalimumab	3 (8.3)	3 (15.0)	0 (0.0)	
Certolizumab pegol	2 (5.6)	0 (0.0)	2 (14.3)	
Etanercept	10 (27.8)	5 (25.0)	5 (35.7)	
Golimumab	5 (13.9)	2 (10.0)	3 (21.4)	
Infliximab	6 (16.7)	4 (20.0)	2 (14.3)	
Non-TNF inhibitors, n (%)				
Abatacept	4 (11.1)	3 (15.0)	1 (7.1)	
Tocilizumab	6 (16.7)	3 (15.0)	1 (7.1)	
**Concomitant csDMARDs, n (%)**				
Methotrexate	25 (69.4)	12 (60.0)	11 (78.6)	0.29^b^
Methotrexate dose (mg/week in users)	8 (7–10)	9 (7–10)	8 (8–10)	0.95^b^
Salazosulfapyridine	13 (36.1)	8 (40.0)	5 (35.7)	1.00^b^
Bucillamine	1 (2.8)	1 (5.0)	0 (0.0)	1.00^b^
Iguratimod	1 (2.8)	1 (5.0)	0 (0.0)	1.00^b^
Leflunomide	1 (2.8)	1 (5.0)	0 (0.0)	1.00^b^
Tacrolimus	1 (2.8)	1 (5.0)	0 (0.0)	1.00^b^
csDMARDs combination	8 (22.2)	5 (25.0)	3 (21.4)	1.00^b^
Concomitant glucocorticoids, n (%)	3 (8.3)	2 (10.0)	1 (7.1)	1.00^b^
Concomitant NSAIDs, n (%)	5 (13.9)	4 (20.0)	1 (7.1)	0.38^b^

Values are presented as median (interquartile range [IQR]) unless otherwise specified.

^a^Mann–Whitney *U* test.

^b^Fisher’s exact test.

*RA* rheumatoid arthritis, *bDMARD* biological disease-modifying antirheumatic drug, *anti-CCP* anti-cyclic citrullinated peptide antibody, *HAQ-DI* Health Assessment Questionnaire-disability index, *CRP* C-reactive protein, *ESR* erythrocyte sedimentation rate, *DAS28* disease activity score 28, *SDAI* simplified disease activity index, *ACR/EULAR* the American College of Rheumatology/the European League Against Rheumatism, *TNF* tumour necrosis factor, *NSAIDs* nonsteroidal anti-inflammatory drugs.

In line with the fact that all enrolled patients fulfilled the SDAI ≤ 3.3 criterion, the medians (IQRs) of TJC28 and SJC were both 0 (0–0) as were those of Health Assessment Questionnaire-disability index (HAQ-DI). The median PtGA (mm VAS) was 3, and the median PhGA (mm VAS) was 0. The median CRP value was 0.04 mg/dL, and the median disease activity score 28—erythrocyte sedimentation rate (DAS28-ESR)^27^ and SDAI were 2.0 and 0.4, respectively. Importantly, 31 (86.1%) of 36 patients had achieved the ACR/EULAR Boolean remission criteria, which have been shown to be the most stringent criteria of clinical RA remission^4^. The ultrasonographic (US) examination of the 40 joints showed that the total power Doppler (PD) score was ‘0’ in 26 patients and ‘1’ in 5 patients. Therefore, imaging remission was confirmed in at least 72.2% of the enrolled patients.

Twenty-six (72.2%) patients had discontinued anti- tumour necrosis factor (TNF) bDMARD, while four and six patients had discontinued the non-TNF abatacept and tocilizumab, respectively. Methotrexate (MTX) was the predominant (69.4%) concomitant csDMARD as an anchor drug for RA in Japan and other countries^1,28^, while concomitant glucocorticoids were rarely administrated to patients (8.3%) who achieved SDAI remission.

### Clinical comparison between patients experiencing RA flare and those maintaining remission

**Figure 1:**
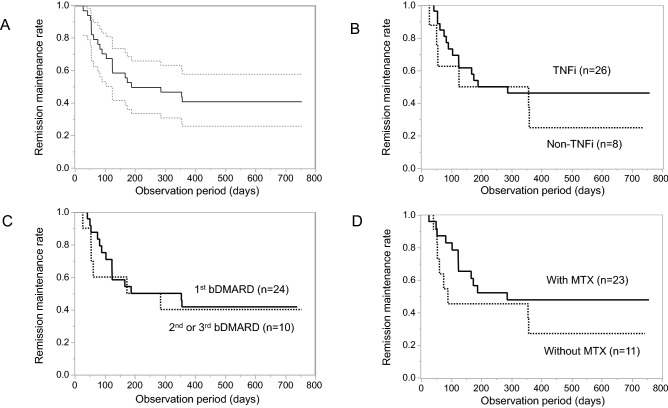
Remission maintenance rate and treatments. (**A**) All patients (n = 36, solid line) with 95% confidence intervals (dotted lines). (**B**) Comparison of patients discontinuing TNFi (n = 26, solid line) and non-TNFi (n = 8, dotted line). (**C**) Comparison of patients discontinuing first bDMARD (n = 24, solid line) and second or third bDMARD (n = 10, dotted line). (**D**) Comparison of patients with concomitant MTX (n = 23, solid line) and without MTX (n = 11, dotted line). *TNFi* tumour necrosis factor inhibitors, *bDMARD* biological disease-modifying antirheumatic drug, *MTX* methotrexate.

Among the 36 enrolled patients, 20 patients (55.6%) experienced RA flare within 2 years, with a range of 43–651 (median 115) days after the first skipped date of bDMARD infusion/injection (Fig. 1A). Notably, 18 (90%) of those patients received the same bDMARD thereafter and again achieved SDAI remission within 2 months. Another bDMARD was successfully introduced for one patient, and the remaining patient achieved SDAI remission after 9 months without treatment adjustment. Thus, the outcome of patients after experiencing disease flare was very favourable. In addition, two patients had not received any csDMARDs at baseline. One patient flared after tocilizumab discontinuation, and another patient did not flare for 2 years after etanercept discontinuation.

The comparison between flare-positive patients (n = 20) and patients who remained in remission (n = 14) is also shown in Table 1. The demographic characteristics were comparable between the two groups. Among the activity-related parameters, the ESR value and DAS28-ESR were numerically greater in flare-positive patients than in flare-negative patients, although the differences were not statistically significant (p = 0.077 and p = 0.058, respectively). US findings were also comparable for the greyscale (GS), PD, and total scores between flare-positive and flare-negative patients (p = 0.54, p = 0.88, and p = 0.53. respectively). Further, the proportion of patients who had a total PD score ≥ 2 was similar between the flare-positive and flare-negative groups (15.0% and 14.3%, respectively; p = 1.00).

As for the RA treatments, disease flare was similarly observed among patients who had discontinued TNF inhibitors versus non-TNF inhibitors, and those who had discontinued the first bDMARD versus the second or third bDMARD (Fig. 1B,C, Table 1). Patients who received MTX showed a trend for maintaining remission compared with those who did not receive MTX (Fig. 1D and Table 1), although the differences were not statistically significant. Therefore, clinical and imaging prediction of disease flare after bDMARD discontinuation in the patients who achieved the stringent criteria of RA remission was not suggested by this study.

### Biomarker comparison between patients experiencing RA flare and those maintaining remission

Next, we compared the baseline plasma concentrations of nine cytokines and three soluble cytokine receptors relevant to the pathogenesis of RA and/or molecular targets of RA treatment (Table 2). Interestingly, although plasma IL-2 concentration was generally very low and undetectable in eight of 36 patients, it was significantly greater in flare-negative patients than in flare-positive patients (p = 0.017). A univariate logistic regression analysis and subsequent receiver operating characteristics (ROC) curve analysis suggested a cut-off value of 0.06287 pg/mL with an area under the curve (AUC) value of 0.74 (Supplementary Fig. 1A).

**Table 2:** Comparison of baseline cytokine and cytokine receptor concentrations between patients who experienced disease flare and those who remained in remission.

	Flare ( +), n = 20	Flare (−), n = 14	p-value
IL-1β	0.032 (0–0.069)	0.051 (0–0.096)	0.39
IL-2	0.056 (0–0.13)	0.17 (0.075–0.30)	0.017
IL-6	0.52 (0.36–2.1)	0.67 (0.41–1.1)	0.73
IL-8	9.2 (6.1–19.6)	14.6 (8.3–20.3)	0.14
IL-10	0.24 (0.14–0.37)	0.26 (0.19–0.40)	0.58
IFNγ	6.3 (2.6–85.4)	7.9 (6.2–14.8)	0.55
TNF	1.9 (1.6–16.9)	4.1 (2.9–33.0)	0.086
GM-CSF	0.068 (0–0.11)	0.12 (0.021–0.31)	0.21
VEGF	12.6 (7.5–18.8)	14.7 (11.5–19.8)	0.29
sTNFR1	1336.5 (1014.6–2055.6)	1184.0 (914.7–1255.8)	0.064
sTNFR2	5701.3 (3720.3–108,451.6)	7017.3 (5457.9–218,396.5)	0.23
sIL-6R	39,382.3 (24,570.5–56,730.8)	47,576.3 (38,110.1–59,504.7)	0.32

Values are median (interquartile range), pg/mL.

*IL* interleukin, *IFNγ* interferon gamma, *TNF* tumour necrosis factor, *GM-CSF* Granulocyte–macrophage colony-stimulating factor, *VEGF* vascular endothelial growth factor, *sTNFR1* soluble tumour necrosis factor receptor 1, *sTNFR2* soluble tumour necrosis factor receptor 2, *sIL-6R* Soluble IL-6 receptor.

Moreover, the Kaplan–Meier analysis showed that lower baseline IL-2 concentration was associated with subsequent disease flare (p = 0.020, Supplementary Fig. 1B).

**Figure 2:**
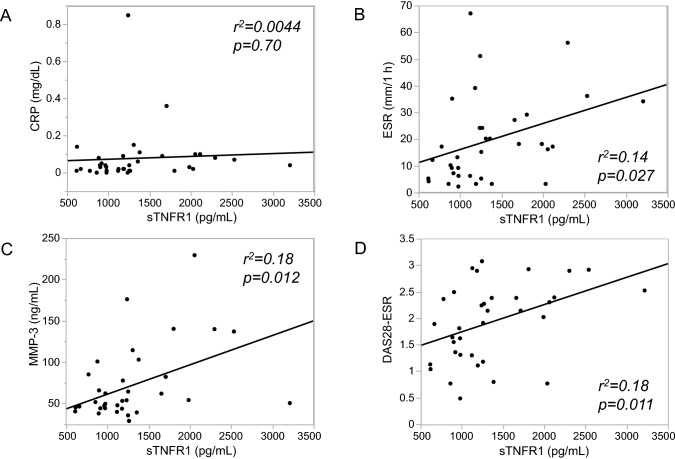
Correlation of plasma sTNFR1 concentration with RA disease activity at baseline (n = 36). The correlation coefficient (r) and p value were determined by Spearman’s rank test. (**A**) CRP; r^2^ = 0.0044, p = 0.70. (**B**) ESR; r^2^ = 0.14, p = 0.027. (**C**) MMP-3; r^2^ = 0.18, p = 0.012. (**D**) DAS28-ESR; r^2^ = 0.18, p = 0.011. *sTNFR1* soluble tumour necrosis factor receptor 1, *RA* rheumatoid arthritis, *CRP* C-reactive protein, *ESR* erythrocyte sedimentation rate, *MMP-3* matrix metalloproteinase-3, *DAS28* disease activity score 28.

Although plasma concentrations of IL-6, TNF, and soluble tumour necrosis factor receptor 2 (sTNFR2) may be affected by some bDMARDs, a marginal difference in soluble tumour necrosis factor receptor 1 (sTNFR1) concentration between flare-positive and flare-negative patients (p = 0.064) led us to further analyses. The plasma sTNFR1 concentration was not correlated with CRP (r^2^ = 0.0044, p = 0.70; Fig. 2A), but it was significantly correlated with ESR (r^2^ = 0.14, p = 0.027; Fig. 2B), matrix metalloproteinase-3 (MMP-3) (r^2^ = 0.18, p = 0.012; Fig. 2C), and DAS28-ESR (r^2^ = 0.18, p = 0.011; Fig. 2D).

**Figure 3:**
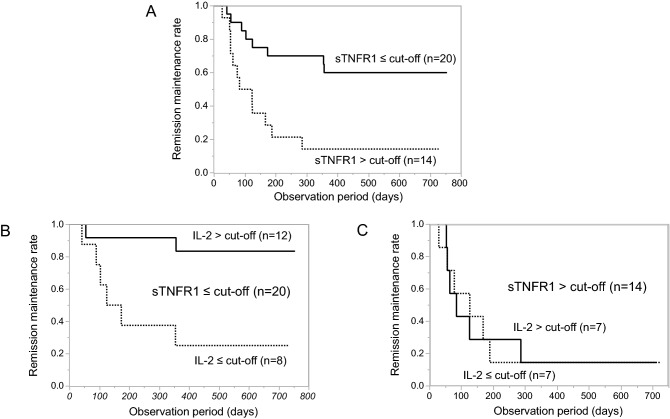
Plasma cytokine profile at baseline as a predictive marker of RA flare after bDMARD discontinuation. (**A**) Comparison of patients with sTNFR1 ≤ cut-off (n = 20, solid line) and sTNFR1 > cut-off (n = 14, dotted line). (**B**) Comparison of patients with IL-2 > cut-off (n = 12, solid line) and IL-2 ≤ cut-off (n = 8, dotted line) in those with sTNFR1 ≤ cut-off (n = 20). (**C**) Comparison of patients with IL-2 > cut-off (n = 7, solid line) and IL-2 ≤ cut-off (n = 7, dotted line) in those with sTNFR1 > cut-off (n = 14). *bDMARD* biological disease-modifying antirheumatic drug, *RA* rheumatoid arthritis, *sTNFR1* soluble tumour necrosis factor receptor 1, *IL* interleukin.

A logistic regression analysis and subsequent ROC analysis suggested a cut-off value of 1267.8 pg/mL with an AUC value of 0.69, and the Kaplan–Meier analysis showed that the baseline sTNFR1 concentration was associated with subsequent disease flare (p = 0.0041, Fig. 3A). The categorical distribution of sTNFR1 was independent of that of IL-2 (p = 0.73), and a higher baseline IL-2 concentration was beneficial for patients with a sTNFR1 concentration below the cut-off value (p = 0.0058, Fig. 3B), but not for those with sTNFR1 concentration above the cut-off value (p = 0.81, Fig. 3C).

## Discussion

This is the first study to comprehensively evaluate patients achieving clinical remission defined by the ACR/EULAR who had discontinued bDMARDs from the clinical, imaging, and biomarker perspectives. Our results indicated the usefulness of combining biomarker, but not clinical and imaging, assessments for the prediction of disease flare referring to the Outcome Measures in Rheumatology (OMERACT) validation study^29^ after bDMARD discontinuation in patients fulfilling the stringent criteria of clinical remission.

In this study, the median baseline values of enrolled patients were ‘0’ for TJC28, SJC28, PhGA (mm VAS), HAQ-DI, and total PD score of 40 joints. Despite such sufficient suppression of disease activity, 20 of 36 patients (55.6%) experienced disease flare within the 2 years of a bDMARDs-free period. Interestingly, baseline US examination and the confirmation of imaging remission added little to the prediction of subsequent RA flare in this study, which enrolled patients who had achieved the stringent criteria for RA remission. This fact is concordant with a recent idea that does not support the routine use of magnetic resonance imaging or US joint examination in the treat-to-target strategy^30^. However, two patients with US PD scores of 22 and 10, experienced disease flare at 55 and 105 days, respectively, after bDMARD discontinuation. These patients had one swollen joint in 28 joints and elevated MMP-3 (229.4 and 176.2 ng/mL, respectively). Therefore, US examination may be useful for patients achieving the stringent remission criteria despite some signs of residual arthritis activity. Nonetheless, it should be noted that imaging remission by US does not guarantee sustained remission after bDMARD discontinuation.

Several previous studies have suggested that longer disease duration, anti-CCP positivity, baseline radiographic damage, and residual disease activity at baseline were associated with disease flare after bDMARD discontinuation^1,31,32^. In fact, disease duration, anti-CCP positivity, and DAS28-ESR were numerically greater in flare-positive patients than in flare-negative patients, although statistically not significant in this study with a small sample size (Table 1). Instead, we identified sTNFR1 and IL-2 as predictive biomarkers of subsequent disease flare. Recently, a sub-analysis of the Reduction of Therapy in Rheumatoid Arthritis Patients in Ongoing Remission study^19^ found that multi-biomarker disease activity (MBDA) at baseline was significantly higher in patients with RA who relapsed than in those remaining in stable remission and those tapering/stopping DMARDs^33^. An MBDA score is calculated by the expression of 12 biomarkers including sTNFR1, IL-6, vascular endothelial growth factor, CRP, and MMP-3 which were also evaluated in this study^34^. The sTNFR1 is generated as a result of proteolytic shedding of TNFR1 by tumour necrosis factor-α-converting enzyme (TACE), a member of the ADAM (a disintegrin and metalloproteinase) family of zinc metalloproteinases^35^. As shown in Fig. 2, sTNFR1 concentration was positively correlated with DAS28-ESR, and the DAS28-ESR value was significantly higher in patients with sTNFR1 above the cut-off value than in those with sTNFR1 below the cut-off value (medians, 2.3 and 1.6, respectively, p = 0.022). These results suggest that sTNFR1 which is incorporated in the MBDA score, is a valid biomarker of RA disease activity even in patients achieving SDAI ≤ 3.3. In addition to the results shown in Fig. 3, a sensitivity analysis in patients discontinuing TNF inhibitors (n = 26) revealed that RA flare was observed in 83.3% (10 of 12) of patients with sTNFR1 above the cut-off value which was significantly greater than the flare rate of 28.6% (4 of 14) patients with sTNFR1 below the cut-off value (p = 0.0079). Furthermore, combining the sTNFR1 and IL-2 biomarkers is promising for the prediction of RA flare after bDMARD discontinuation (Fig. 2). Although the blood concentration of IL-2 was also found to be very low (approximately 1 pg/mL) in several other studies of non-rheumatic diseases^36–38^ and mostly below the lower limit of detection in a very recent study of predicting drug-free RA remission by a composite score incorporating three transcripts, IL-27, and Boolean remission^39^, and thus only detectable using high-sensitivity measurements, IL-2 is crucial for the development and expansion of regulatory T cells^40,41^ which may be associated with sustained RA remission.

The limitations of this study include the small sample size from a single centre and the lack of histological or cellular analyses. However, the comprehensive and intensive evaluations from multiple aspects including clinical, imaging and biomarker analyses are the strengths of the study.

In conclusion, this was the first study to identify the usefulness of a combined biomarker evaluation of sTNFR1 and IL-2 for the prediction of sustained remission for 2 years after bDMARD discontinuation in patients with RA. Recently, the difference between clinical remission and ‘molecular remission’ has been proposed by multi-omics monitoring of responses to bDMARDs in patients with RA^42^. Further analyses focusing on the next objectives including earlier detection of RA flare by comprehensive evaluations and the elucidation of relationships among clinical, imaging, and biomarker changes are ongoing in our laboratory.

## Methods

### Patients

Patients with RA who visited Toho University Ohashi Medical Center in Tokyo, Japan, between November 2014 and January 2018 were eligible for the Flare Landscape from clinical, imaging, and biomarker Aspects In Rheumatoid arthritis (FLAIR) study when they met all of the following criteria: (1) fulfilling the 2010 ACR/EULAR classification criteria for RA^43^; (2) maintaining ACR/EULAR clinical remission defined by SDAI ≤ 3.3 for at least 3 months on ≥ 2 visits; (3) being willing to discontinue an ongoing bDMARD with consideration of the risk–benefit balance of bDMARD discontinuation.

### Study design

This was a 2-year, prospective, observational study. The primary endpoint was flare DAS28-ESR ≥ 3.2 with an increase from the baseline, > 0.6^29^) within 2 years after bDMARD discontinuation. The study protocol was approved by the Ethics Committee of Toho University Ohashi Medical Center (Ohashi approval number, 14–9), and this study was carried out in compliance with the Declaration of Helsinki. Written informed consent was obtained from all participants.

The target sample size was set to 40 patients to obtain data of 20 patients experiencing disease flare for intensive analyses of clinical, imaging, and biomarker changes estimating a 50% flare rate. Patient recruitment was started in November 2014 and closed in January 2018 with the enrolment of 36 patients.

### Clinical assessments

We obtained the following medical information from medical records: age, sex, height, weight, disease duration from the onset of symptoms to enrolment, duration of bDMARD treatment to be discontinued, remission duration, Steinbrocker radiographic stage, anti-CCP, rheumatoid factor, MMP-3, and the treatment for RA. After enrolment in this study, the patients were examined every 2–3 months for TJC28, SJC28, PtGA, PhGA, HAQ-DI, blood tests for ESR, serum CRP, and MMP-3 as well as plasma storage for future examination of cytokine and soluble cytokine receptor concentrations, and also underwent US examination of the 40 joints.

### US examination

A Xario (Toshiba Medical Systems, Tochigi, Japan) US machine equipped with a multifrequency linear array probe (7–14 MHz) was used. The PD settings were as follows: PD pulse repetition frequency, 16.5 kHz (flow range, 3.8 cm/s); Doppler frequency, 6.1 MHz, and low wall filter. Colour gain was set just below the level at which noise appeared. The US examination was performed according to the EULAR guidelines for musculoskeletal US in rheumatology^44,45^ by rheumatologists and ultrasonographers certified by the Japan College of Rheumatology. These examiners evaluated 40 of the joints including the bilateral shoulders, elbows wrists, metacarpophalangeal joints, thumb interphalangeal joints, second through fifth finger proximal interphalangeal joints, knees, ankles, and metatarsophalangeal joints using longitudinal and transverse scans, without knowledge of the other medical information of the patients.

The US examiners performed the final scoring of the recorded US findings according to the OMERACT definitions^46^. Briefly, the GS was graded semiquantitatively from 0 to 3 (0 = absent, 1 = mild, 2 = moderate, and 3 = marked) in a combined measure of synovial hypertrophy and fluid retention of the articular recess. The intra-articular PD signals were also graded on a 0–3 scale, and the total joint US score was determined by summing all GS and PD scores for each patient.

### Measurement of plasma concentration of cytokines and soluble cytokine receptors

Plasma was stored at − 80 °C until measurement. Plasma concentrations of interleukin (IL)-1β, IL-2, IL-6, IL-8, IL-10, interferon gamma, TNF, granulocyte–macrophage colony stimulating factor, vascular endothelial growth factor, sTNFR1, sTNFR2, and soluble IL-6 receptor were measured by electrochemiluminescence assay with the Ultra-Sensitive Kit (Meso Scale Discovery, Rockville, MD, USA) according to the manufacturer’s protocol^47^.

### Statistical analyses

The statistical analyses were performed using JMP Pro (version 14.2, SAS Institute Japan Ltd., Tokyo, Japan). Continuous variables are presented as the median and interquartile range (IQR) and were analysed using the Mann–Whitney *U* test. Binomial data were compared between two groups using Fisher’s exact test. Kaplan–Meier analysis and log-rank tests were used for the comparison of survival curves between the groups. The correlation coefficient (r) was determined by Spearman’s rank test. Logistic regression analyses followed by ROC curve analyses were conducted for the prediction of RA flare based on the baseline biomarkers. p-values < 0.05 were considered statistically significant.

## Supplementary Information

Supplementary Information
