# Supplementary material for: Prediction of disease flare by biomarkers after discontinuing biologics in patients with rheumatoid arthritis achieving stringent remission
Source: Sci Rep. 2021 Mar 25;11:6865. doi: 10.1038/s41598-021-86335-7 (PMC7994312; doi:10.1038/s41598-021-86335-7)
Supplement: Supplementary file 1 — Supplementary Information [file 41598_2021_86335_MOESM1_ESM.docx]

**Prediction of disease flare by biomarkers after discontinuing biologics in patients with rheumatoid arthritis achieving stringent remission**

Hideto Kameda, Ayako Hirata, Takaharu Katagiri, Yuto Takakura, Yuki Inoue, Sayaka Takenaka, Hideki Ito, Kennosuke Mizushina and Takehisa Ogura

Division of Rheumatology, Department of Internal Medicine, Toho University, Tokyo, Japan

Corresponding Author:

Address correspondence and reprint request to Hideto Kameda, Professor of Medicine, Division of Rheumatology, Department of Internal Medicine, Toho University Ohashi Medical Center, 2-22-36 Ohashi, Meguro-ku, Tokyo 153-8515, Japan

Tel: +81-3-3468-1251; Fax: +81-3-5433-3069

E-mail: hideto.kameda@med.toho-u.ac.jp

ORCID: 0000-0002-4330-5782

**Supplementary Figure 1.**

**Plasma IL-2 concentration at baseline as a predictive marker of RA flare after bDMARD discontinuation.**

A. ROC curve of plasma IL-2 concentration for RA flare prediction.

B. Comparison of patients with IL-2 > cut-off (n=19, solid line) and IL-2 ≤ cut-off (n=15, dotted line)
